# Impact of Work–Family Balance on Nurses’ Perceived Quality of Life During the COVID-19 Pandemic: A Scoping Review

**DOI:** 10.3390/nursrep14040294

**Published:** 2024-12-14

**Authors:** Ana María Antolí-Jover, María Gázquez-López, Pascual Brieba-del Río, María Ángeles Pérez-Morente, Adelina Martín-Salvador, María Adelaida Álvarez-Serrano

**Affiliations:** 1 Department of Nursing, Faculty of Health Sciences in Ceuta, University of Granada, 51001 Ceuta, Spain; antolijover@ugr.es (A.M.A.-J.); adealvarez@ugr.es (M.A.Á.-S.); 2 Instituto Nacional de Gestión Sanitaria, 51002 Ceuta, Spain; pbrieba@ingesa.sanidad.gob.es; 3 Department of Nursing, Faculty of Health Sciences, University of Granada, 18016 Granada, Spain; ademartin@ugr.es

**Keywords:** work–family balance, quality of life, nursing, COVID-19

## Abstract

Background/Objectives: The COVID-19 pandemic has put nurses under extreme pressure, especially affecting them by significantly increasing their workload and compromising their well-being. The lack of balance between work and personal life has caused greater stress and burnout in these professionals, deteriorating their quality of life and the care they provide. This study aims to synthesize the scientific evidence on the relationship between work–family balance and the perceived quality of life of nurses during the COVID-19 pandemic. Methods: A scoping review was carried out based on the Joanna Briggs Institute methodology, following the PRISMA-ScR guidelines in the PubMed, CINAHL, SCOPUS, WOS, Cochrane Library, and PsycINFO databases. The quality of the articles was assessed using the Mixed Methods Appraisal Tool (MMAT). Results: Five studies involving 1641 nurses from Spain, India, Jordan, South Korea, and Turkey focused on three key areas: (1) work–life balance, (2) quality of life, and (3) factors associated with quality of life during the COVID-19 pandemic. Conclusions: The pandemic exacerbated pre-existing challenges related to work–life balance and quality of life, particularly for nurses with rigid and extended work hours, increasing stress and negatively impacting their well-being. The study emphasizes the need for flexible labor policies, psychological support, and strong social networks to prevent burnout and improve nurses’ quality of life.

## 1. Introduction

The global COVID-19 pandemic has generated an unprecedented crisis that has deeply impacted healthcare systems and the professionals who work within them, especially nurses, who are on the front lines of patient care [1,2,3]. The impact of the pandemic on nurses’ physical and emotional health, as well as their ability to balance work and personal life demands, has become an increasingly important topic in recent years, as these professionals have faced extreme working conditions [4,5]. In this context, quality of life (QoL) and work–life balance (WLB) have been seriously compromised, which has implications for both their personal well-being and the quality of care they provide to patients [6,7].

Health-related quality of life (HRQoL) and work-related quality of life (QoWL) are two essential dimensions for understanding nurses’ well-being [8]. HRQoL encompasses an individual’s overall perception of well-being, influenced by their physical and mental health, while QoWL focuses on professionals’ satisfaction with their perceived stress or burnout, without comprehensively exploring how the pandemic has changed working conditions, physical and mental health, and nurses’ perceptions of their overall well-being [9,10]

This study is positioned within this gap in the literature, focusing exclusively on the effects of the pandemic on nurses’ QoL and WLB. By analyzing how structural changes brought about by the health crisis have affected the well-being of this group, it aims to provide a deeper understanding of the risk factors and working conditions nurses have had to face. Through this analysis, the goal is to highlight how these conditions impact not only the health of nurses but also the quality of care they provide to patients, which has implications both individually and systemically [11].

One key point emerging from this analysis is the relationship between nurses’ professional burnout and patient safety. When nurses face significant imbalances between their personal and professional lives, the direct result is a decrease in the quality of care provided, which can lead to adverse consequences for patient health, such as an increase in medical errors, morbidity, and hospital mortality. This phenomenon not only affects patients immediately but can also have long-term effects on the sustainability of healthcare systems [12,13].

Therefore, the primary objective of this study is to thoroughly analyze how the pandemic has affected nurses’ working conditions and their ability to manage the demands of both their professional and personal lives [14]. Although both dimensions are closely interrelated, the existing literature has tended to address them separately, limiting a holistic understanding of the factors affecting nurses’ well-being. In particular, the COVID-19 pandemic has drastically altered both dimensions, as nurses have had to face longer work hours, more demanding working conditions, and a significant increase in emotional and physical demands [15].

Before the pandemic, it was already known that nurses faced significant difficulties balancing their work responsibilities with their personal needs [16]. Factors such as emotional exhaustion, chronic stress, and physical fatigue were common due to the high demand for healthcare services, staff shortages, and inadequate shift organization [17,18]. However, with the onset of the global health crisis, these factors were alarmingly exacerbated [2,19]. Nurses faced a dramatic increase in workload, a greater level of exposure to infection, and the management of a growing number of patients in emergency situations. This overload not only affected their physical and mental health but also had a significant impact on their ability to balance their work life with their family responsibilities, leading to a deterioration in their WLB [20,21].

In this context, the scientific literature on the impact of the pandemic on nurses’ well-being is still in its early stages. Although some studies have analyzed the immediate effects of the pandemic, such as increased stress and burnout, there is a lack of systematic studies that delve into how the crisis has altered nurses’ QoL and WLB in different healthcare contexts. Most available studies have focused on narrow aspects, such as perceived stress or burnout, without comprehensively exploring how the pandemic has altered working conditions, physical and mental health, and nurses’ overall perception of well-being [22,23]. In doing so, it aims to identify the main areas of vulnerability and risk factors and offer recommendations for the design of preventive strategies that not only mitigate the effects of future crises but also improve nurses’ working conditions in their daily lives. This study has implications for both occupational health and public health policy, as the health and well-being of nurses are key elements in ensuring quality patient care.

## 2. Materials and Methods

### 2.1. Study Design

A scoping review was conducted following the methodology proposed by the Joanna Briggs Institute for scoping reviews, and its writing adhered to Preferred Reporting Items for Systematic Reviews and Meta-Analyses for Scoping Reviews (PRISMA-ScR) [24] guidelines to assess the evidence available up to 24 July 2024. For this review, the databases consulted were PubMed, CINAHL, SCOPUS, WOS, Cochrane Library, and PsycoINFO.

In order to identify the most relevant keywords for the search, the Health Sciences Descriptors Thesaurus (DeCS) was used. This set of controlled terms was applied to all the databases, ensuring an exhaustive and consistent search in each of them.

Boolean operators were used in the search to effectively refine the results. “AND” and “OR” were used to combine terms and broaden the thematic search.

Thus, the following algorithm was designed: ((Work-Life Balance OR Work-Life Conflict OR Conflict, Work-Life OR Conflicts, Work-Life OR Work Life Conflict OR Work-Life Conflicts OR Work-Family Balance OR Balance, Work-Family OR Balances, Work-Family OR Work Family Balance OR Work-Family Balances OR Life-Work Imbalance) AND (Nurses OR Nurse OR Personnel, Nursing OR Nursing Personnel OR Registered Nurses OR Nurse, Registered OR Nurses, Registered OR Registered Nurse) AND (Quality of Life OR Life Quality OR Health-Related Quality Of Life OR Health Related Quality Of Life OR HRQOL)).

The inclusion criteria applied required the studies to be original (descriptive, cross-sectional, cohort, case-control, and qualitative studies, as well as randomized clinical trials) and published in any language. Studies that were published from the start of the COVID-19 pandemic between January 2020 and July 2024 were included. Studies from before the pandemic, those with a sample comprising nursing students, those that did not address QoL and WLB, and those that did not allow full access to the text of the article were excluded.

### 2.2. Data Collection

For the collection and organization of data, an Excel spreadsheet and the bibliographic manager Zotero were used. In the first instance, the data from the selected databases were recorded, including the title of the article, the authors, and the year of publication. This initial step focused on identifying and eliminating duplicates, thus ensuring the uniqueness of the studies considered.

The selection of the articles was carried out in several phases. In the first phase, the title and abstract of each article were read, applying predefined inclusion and exclusion criteria. Only those articles that met these criteria advanced to the next phase of selection. In the second phase, the articles that passed the first screening were evaluated by reading the entire text, selecting only studies that were carried out from the beginning of the COVID-19 pandemic and that studied WLB and its influence on nurses’ QoL.

The quality of the selected studies was assessed using the MMAT (Mixed Methods Appraisal Tool) [25]. This tool is specifically designed for systematic reviews that include quantitative, qualitative, and mixed studies. Each study was assessed using seven items: two items common to all study designs and five items specific to each type of design (quantitative, qualitative, or mixed). The possible answers for each item were “Yes”, “No”, and “I don’t know”. Based on the affirmative (“Yes”) answers obtained in the assessment items, a quality percentage was calculated for each study.

The process of selecting and assessing the articles was carried out by two lead authors. To resolve any discrepancies that arose during the process, a third author intervened as a mediator, thus ensuring objectivity and consistency in the assessment. The application of a methodical and rigorous approach not only guarantees the reliability and validity of the scoping review, but also provides an objective and comprehensive assessment of the quality of the selected studies.

The synthesis of these data was carried out with the aim of offering a clear and coherent view of the most relevant findings. This process allowed for the information to be consolidated, thus facilitating a comprehensive and detailed understanding of the studies in relation to the objectives established in the review.

## 3. Results

**Figure 1:**
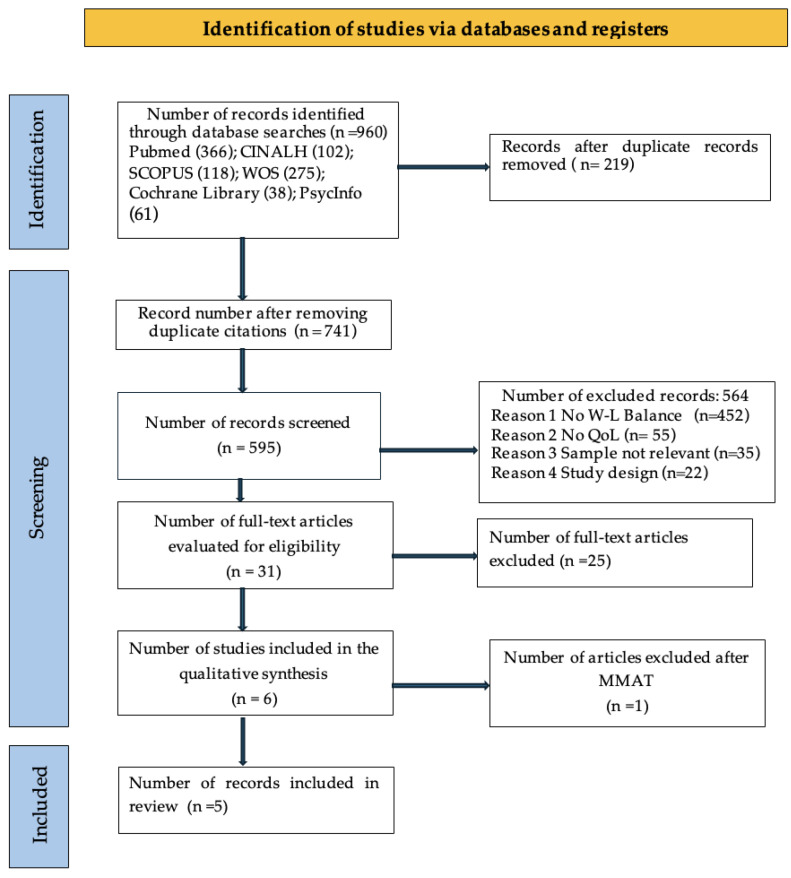
Flowchart made after the query was carried out for all databases.

The complete process for searching and selecting the studies included in this scoping review is presented in the flowchart in Figure 1 below, following PRISMA standards.

### 3.1. Assessment of Methodological Quality

After applying the MMAT tool (Table 1), the articles by Antolí et al. [26], Al-Hammouri et al. [27], and You [28] obtained 100% affirmative responses in all items. The articles by Nanjundeswaraswamy et al. [29] and Daşbilek et al. [30] achieved 80% positive responses.

In the study by Lorber et al. [31], 40% of the responses were affirmative. This score is due to the fact that the answers to items 4.2 and 4.4 were “I don’t know”. This is because it could not be guaranteed that the sample was representative of the target population (item 4.2) or that the risk of non-response bias was low (item 4.4). Regarding item 4.3, which states that the measures are appropriate, the answer was “No”.

### 3.2. Characteristics of the Studies

After the screening and suitability phase, six articles were included. Next, the methodological quality was assessed, developed by Hong et al. [25], and one of them was rejected. As a result, a sample of five descriptive quantitative articles was established. The number of nurses participating in these studies was 1641, and the studies were conducted in Spain, India, Jordan, Korea, and Turkey.

The main characteristics of the included articles are described in Table 2.

### 3.3. Summary of Results

The reviewed studies address three main topics: WLB, nurses’ QoL, and factors associated with QoL.

#### 3.3.1. Work–Life Balance (WLB) Outcomes

The results related to the balance between family and work life can be seen in Table 2. The five studies present different views on the balance between work and family. Nanjundeswaraswamy et al. [29] and You [28] analyzed this topic globally, while Al-Hammouri et al. [27] and Daşbilek et al. [30] focused on conflicts between both areas. Antolí et al. [26] examined both positive and negative interactions between family and work life.

On the other hand, Antolí et al. [26] examined both positive and negative interactions between family and work life using the Work–Family Interaction Questionnaire (SWING) [33]. Scores on this instrument range from zero to three and are categorized as follows: scores between zero (inclusive) and less than one indicate “low interaction”, scores between one (inclusive) and less than two indicate “medium interaction”, and scores between two (inclusive) and three indicate “high interaction”. The results showed mean scores of 1.24 ± 0.519 for Negative Work–Family interaction, 0.46 ± 0.443 for Negative Family–Work interaction, 1.44 ± 0.634 for Positive Work–Family interaction, and 1.95 ± 0.693 for Positive Family–Work interaction, indicating varying levels of interaction across these dimensions.

Al-Hammouri et al. [27] used the Work and Family Conflict Scale (WAFCS) to assess bidirectional conflicts between work and family (from work to family and from family to work). The scale scores for both subscales range from 5 to 35, with higher scores indicating greater levels of conflict. The results reported by the authors show an average work-to-family conflict score of 16.31 ± 6.17 and a family-to-work conflict score of 23.6 ± 6.91, highlighting a greater impact of the family domain on the work domain.

Daşbilek et al. [30] also used the Work Life Balance Scale (WLBS) as a measurement instrument; however, they focused on work-to-family conflict and family-to-work conflict rather than assessing overall work–life balance. However, this is a five-point Likert scale, in which one means “definitely disagree” and five means “definitely agree”. The minimum score is 10 and the highest is 50. A higher score indicates the existence of conflict between work and family. The results reported by the authors indicate a work-to-family conflict score of 20.282 ± 2.909 and a family-to-work conflict score of 18.598 ± 4.504.

You [28] used the WLBS to analyze WLB globally. The scale used was developed by Kim and Park [36] and consists of 29 questions: eight items on the level of harmony between work and family, eight items on the level of harmony between work and leisure, nine items on the level of harmony between work and personal growth, and four items on general life evaluations. It is a Likert scale ranging from zero to six points, in which the minimum score is zero and the maximum is one hundred seventy-four. The results are then normalized to obtain final scores between zero and six, with higher scores indicating a greater level of harmony between work and family. The results obtained were 2.43 ± 1.02.

Nanjundeswaraswamy et al. [29] developed an instrument to measure WLB based on a literature review. They defined the construct around three dimensions: flexible work timing, vacation policy, and shift methods. Each of these dimensions was assessed using three items on a five-point scale, in which higher scores indicated a better work–life balance. The instrument was applied during two periods, with an average score of 4.18 ± 0.616 in the pre-pandemic period and 3.40 ± 0.482 during the COVID-19 pandemic.

#### 3.3.2. Nurses’ QoL

Regarding QoL, two types are distinguished: HRQoL [19] and QoWL [20,21,22,23]. The results are detailed in Table 2.

Antolí et al. [26] used the European Quality of Life Questionnaire with five dimensions (EQ-5D index) to assess key aspects of QoL. This tool evaluates mobility, self-care, daily activities, pain/discomfort, and anxiety/depression. The EQ-5D index ranges from one (optimal health) to zero (death). Additionally, the questionnaire includes the Visual Analogue Scale (EQ-VAS), which measures the individual’s perception of their health on a scale from zero to one hundred, with zero representing the worst imaginable health state and 100 the best imaginable health state. The results obtained were an EQ-5D index of 0.820 (S.D. of 0.154) and an EQ-VAS index of 74.56 (S.D. of 15.735).

Regarding QoWL, Al-Hammouri et al. [27] utilized the WRQoL questionnaire, which evaluates workplace conditions, personal and organizational factors, and their impact on employees’ QoL. In the study, QoWL scores ranged from 1.34 to 4.95, in which 1.34 represents a low QoWL and 4.95 represents a high QoL. The mean QoWL score was 3.03 ± 0.75 out of 5, reflecting a moderate-to-positive perception of QoWL of among the participants.

Nanjundeswaraswamy et al. [29] measured the level of QoWL of nurses using Swamy et al.’s instrument [38]. This instrument consists of nine components: work environment, organization culture and climate, relation and cooperation, training and development, compensation and rewards, facilities, job satisfaction and job security, autonomy of work, and adequacy of resources. Nanjundeswaraswamy et al. [29] added two more components: stress and WLB. This modified instrument was validated, achieving a Cronbach’s alpha of 0.97. They evaluated the level of QoWL before and during the pandemic, using an estimated regression equation, in which the mean values of individual components were substituted out. The resulting value corresponds to a five-point scale. The estimated regression equation revealed that nurses’ QoWL scores were 4.36 pre-pandemic and 3.35 during the COVID-19 pandemic on a five-point scale. The chi-square test results for work–life quality showed a significant difference in satisfaction levels before and during the pandemic, with a *p*-value of 0.02. These findings suggest a significant association between the pandemic and nurses’ satisfaction with their QoWL.

Daşbilek et al. [30] used the Turkish-validated version of the Professional Quality of Life Scale (ProQoL) as a measurement tool to assess professional QoL. This scale evaluates average scores across three dimensions: compassion satisfaction, burnout, and secondary traumatic stress (STS). The results of the ProQoL are interpreted within three scoring ranges: low (scores ≤ 22), moderate (scores between 23 and 41), and high (scores ≥ 42). The results obtained in the three dimensions showed scores of 28.58 ± 5.7 in burnout, 27.19 ± 6.54 in STS and 32.95 ± 6.17 in compassion satisfaction.

Finally, You [28] utilized the Korean-validated version of the ProQoL. The study reported mean scores of 2.89 ± 0.64 for compassion satisfaction, 2.96 ± 0.49 for burnout, and 2.58 ± 0.59 for STS. The ProQoL uses a scoring range from one to five, with five indicating the highest level of professional QoL.

#### 3.3.3. Factors Associated with QoL Outcomes

Regarding the factors associated with QoL, studies have identified several elements that influence the well-being of nursing professionals, as detailed in Table 3. Among the sociodemographic factors, gender, educational level, living with a partner, having children, and having paid caregiving support stand out.

In terms of gender, You [28] found that women reported higher levels of burnout (2.97 ± 0.48; *p* = 0.029) and STS (2.60 ± 0.57; *p* = 0.011) compared to men (burnout: 2.59 ± 0.61; STS: 2.06 ± 0.79). Similarly, Daşbilek et al. [30] reported that women had higher scores in burnout (28.945 ± 5.686; *p* = 0.032), compassion satisfaction (33.677 ± 6.046; *p* < 0.001), and STS (27.640 ± 6.543; *p* = 0.022).

Educational level also showed significant differences in both burnout and STS. Daşbilek et al. [30] found that professionals with postgraduate studies had higher burnout scores (28.960 ± 5.484) than university graduates (28.098 ± 4.760) and college graduates (26.500 ± 7.911), with significant differences between postgraduate and college levels (*p* = 0.011). In STS, university graduates (27.390 ± 6.085) and postgraduates (27.531 ± 6.199) also reported higher scores than college graduates (24.300 ± 8.835), with significant differences between these groups (*p* < 0.01). You [28] also observed that participants with higher education levels had higher STS scores, with significant differences between university and postgraduate levels (*p* = 0.011).

Living with a partner was associated with a better QoL, both in the EQ-5D index (ß = 0.174; *p* < 0.05) and the EQ-5D VAS (ß = 0.16; *p* < 0.05), according to Antolí-Jover et al. [26]. Having children was associated with higher levels of burnout (29.489 ± 5.359 vs. 28.050 ± 5.831; *p* = 0.006) and compassion satisfaction (33.844 ± 6.627 vs. 32.436 ± 5.837; *p* = 0.013), while the number of children negatively affected the EQ-5D index (ß = −0.146; *p* < 0.05). Additionally, having paid caregiving support was positively associated with QoL in both the EQ-5D index (ß = 0.149; *p* < 0.05) and the EQ-5D VAS (ß = 0.18; *p* < 0.05).

Regarding occupational factors, shift type, working conditions, work environment, compensation, organizational culture, colleague relationships, professional development, and job tenure are key factors. Antolí-Jover et al. [26] found that workers with changing shifts had a higher EQ-5D index (ß = 0.158; *p* < 0.05). Al-Hammouri et al. [27] reported higher scores for those working fixed shifts (3.23 vs. 2.83; *p* = 0.004), similar to You’s results [28], which also showed lower burnout levels in fixed shifts (2.81 ± 0.42) compared to changing shifts (3.00 ± 0.50; *p* = 0.017).

Working conditions and the work environment also influence QoL. Nanjundeswaraswamy et al. [29] found that factors such as working conditions (ß = 0.209; *p* = 0.004), the work environment (ß = 0.160; *p* = 0.025), compensation (ß = 0.200; *p* = 0.002), and organizational culture (ß = 0.235; *p* = 0.001) positively impacted QoL. Relationships among colleagues (ß = 0.171; *p* = 0.011) and professional development (ß = 0.196; *p* = 0.004) also showed positive associations, especially during the pandemic.

Regarding WLB, it was found that a good balance between work and personal life positively impacts QoL. Antolí-Jover et al. [26] noted that a positive work–family balance improves the EQ-5D VAS score (ß = 0.218; *p* < 0.05), while work–family conflict decreases both the EQ-5D index (ß = −0.399; *p* < 0.05) and the EQ-5D VAS score (ß = −0.337; *p* < 0.05). Al-Hammouri et al. [27] also observed a significant reduction in QoL with work–family conflict (r = −0.49; *p* = 0.01) and family–work conflict (r = −0.20; *p* = 0.01). During the pandemic, Nanjundeswaraswamy et al. [29] found that a better WLB (ß = 0.251; *p* = 0.001) improved one’s QoL. You [28] showed that a good WLB reduced burnout (ß = −0.68; *p* < 0.001), increased compassion satisfaction (ß = 0.42; *p* < 0.001), and decreased STS (ß = −0.56; *p* < 0.001).

In terms of health status, You [28] reported significant differences in burnout, compassion satisfaction, and STS based on perceived health. Participants with poorer health showed higher burnout levels (3.30 ± 0.57) compared to those with regular (3.05 ± 0.426) or good health (2.57 ± 0.40; *p* < 0.001). For compassion satisfaction, those reporting good health scored higher (3.21 ± 0.63) than those in regular (2.81 ± 0.56) or poor health (2.55 ± 0.87; *p* < 0.001). Similarly, in STS, those in poorer health showed higher scores (3.04 ± 0.5) compared to those in regular (2.65 ± 0.50) and good health (2.22 ± 0.59; *p* < 0.001).

## 4. Discussion

This study aimed to synthesize the available scientific evidence on the WLB of nursing professionals during the COVID-19 pandemic and its relationship with perceived QoL. The relevance of this topic lies in its direct impact on the quality of care that professionals provide to patients, as indicated by previous research [39,40]. However, after conducting the search, only five articles addressing this issue were selected, four of which were developed in Asia [27,28,30] and one in Europe [26]. The lack of evidence from other regions of the world limits our ability to compare the experiences of nursing professionals and underscores the need to expand research in this area, particularly in the context of future pandemics.

Within the framework of the studies reviewed, three main areas were identified for analyzing the topic: WLB, QoL, and the factors associated with QoL. Each of these aspects reveals important implications for clinical practice and the well-being of professionals.

### 4.1. Work–Life Balance (WLB)

WLB has gained significant relevance in health organization studies, particularly concerning nurses. The conflict or imbalance between these two domains has been shown to generate greater levels of stress and bidirectional burnout. Traditionally, research has focused on conflicts arising when work responsibilities interfere with family life and vice versa [41]. However, the studies reviewed provide diverse results on this aspect.

The studies by Al-Hammouri et al. [27] and Daşbilek et al. [30] highlight significant conflicts between work and family life, especially among professionals with rotating work schedules. This situation reflects the inherent difficulty of reconciling work and family responsibilities, particularly when schedules are unpredictable and work demands are intense. The prevalence of these conflicts, both from work to family and from family to work, emphasizes the negative impact of rigid and prolonged shifts on the well-being of professionals. The lack of flexibility in work schedules is consistently associated with increased stress levels, affecting QoL and contributing to emotional burnout.

In contrast to the results focused on the negative aspects of WLB, some studies suggest that work–family interactions are not always in conflict. The findings of Antolí et al. [26] show that positive interactions may predominate in some cases. With scores indicating a moderate positive interaction between work and family, these results suggest that one’s work can have beneficial effects on one’s personal life, facilitating greater synergy between both domains. This highlights the importance of adopting a comprehensive approach to WLB that not only considers the negative aspects but also values the positive influences arising from the integration of work and family roles, providing a more complete and accurate view of the factors affecting the balance between them.

Moreover, studies comparing pre-pandemic and pandemic WLB offer a key perspective on how extraordinary events like COVID-19 affect this balance. In this regard, the findings of Nanjundeswaraswamy et al. [29] show a significant decline in WLB during the pandemic, with scores dropping from 4.18 ± 0.616 before the crisis to 3.40 ± 0.482 during it. This decline highlights how the pandemic not only exacerbated pre-existing problems but also introduced new challenges, particularly in high-demand sectors such as healthcare. Healthcare professionals faced increased workloads, longer shifts, and the restructuring of their routines, making it even more difficult to reconcile work and family responsibilities.

It is noteworthy that the studies by Daşbilek et al. [30] and You [28] employ the Work–Life Balance Scale (WLBS) with different methodological approaches and objectives, highlighting differences in the measurement of the WLBS. Daşbilek et al. [30] used a version focused on bidirectional conflict between work and family, specifically assessing tensions affecting both domains. In contrast, You [28] used a broader scale developed by Kim and Park, designed to analyze WLB from a holistic and positive perspective, encompassing harmony in areas such as work–family, leisure, and personal growth. While Daşbilek et al.’s approach identifies imbalances through conflict [30], You’s perspective provides a holistic view based on harmony and global satisfaction, offering complementary perspectives on WLB [28].

Nanjundeswaraswamy et al. [29] introduce a new methodological perspective by defining WLB through specific dimensions such as flexible working hours, vacation policies, and shift methods. These dimensions are assessed using specific items on a five-point scale, providing a practical and adaptable approach to organizational contexts, which also allows for observing how circumstances such as the COVID-19 pandemic can influence WLB. Together, these studies emphasize how different approaches and constructs can complement the understanding of WLB, expanding its conceptual and methodological scope.

This pattern of WLB deterioration during the pandemic is consistent with other studies documenting increased stress and burnout in the healthcare sector [42,43], underscoring the urgent need to revise labor policies and support for healthcare workers. The health crisis amplified workers’ difficulties in effectively managing both their work and family responsibilities, and the results confirm that this deterioration in WLB was not only a consequence of the crisis but also an indicator of the growing pressure on healthcare personnel in times of emergency.

### 4.2. Quality of Life (QoL)

The QoL of nursing professionals is a central topic that affects both their well-being and the quality of care they provide to patients [44,45]. The studies analyzed reveal a diversity of approaches and measurements that enhance our understanding of this concept. While the EQ-5D index used by Antolí et al. [26] focuses on HRQoL and primarily measures physical and emotional health, the WRQoL scale employed by Al-Hammouri et al. [27] centers on QoWL, evaluating working conditions and their impact on professionals’ well-being. Additionally, other studies, such as those by Daşbilek et al. [30] and You [28], have used the ProQoL scale to measure emotional aspects such as burnout and professional satisfaction, offering a comprehensive perspective that encompasses the health, working conditions, and emotional well-being of nurses. This variety of approaches emphasizes that QoL is a multidimensional concept that must be addressed from different perspectives, as physical health, working conditions, and emotional well-being are interconnected and mutually influential. However, this diversity in the instruments used poses the challenge of establishing a common framework that facilitates comparisons across different geographical and cultural contexts.

A significant finding in the studies analyzed is the substantial impact the COVID-19 pandemic has had on nurses’ QoL, especially regarding their work-related well-being. In the study by Antolí et al. [26], the pre-pandemic HRQoL index in Spain showed a positive perception of nurses’ health (0.820 out of 1.0). However, this value does not necessarily reflect a global reality, as working conditions and healthcare systems vary significantly across countries. In some regions, such as developing countries, working conditions are much more challenging, which can negatively impact nurses’ QoL [46]. On the other hand, Asian studies present a different view. Al-Hammouri et al. [27], using the WRQoL scale, reported a moderate perception of QoWL (3.09 out of 5), indicating that although nurses experience an acceptable level of work-related well-being, there are still areas that require improvement, such as WLB and schedule flexibility. These results highlight that while the work situation may be acceptable in some regions, significant challenges persist that affect the well-being of professionals.

Regarding the effects of the pandemic, Nanjundeswaraswamy et al. [29] observed a significant decrease in nurses’ QoWL scores, which dropped from 4.36 before the pandemic to 3.35 during the health crisis. This decline underscores the negative impact of the pandemic on nurses’ working conditions, highlighting how factors such as extreme stress, workload overload, and lack of resources contributed to a considerable decrease in their job satisfaction. This finding is crucial as it shows how a global event can drastically alter healthcare workers’ well-being and underscores the urgent need to improve working conditions, especially in times of crisis.

In addition to the global analysis, the studies by Daşbilek et al. [30] and You [28] document moderate levels of burnout and STS among nurses during the pandemic, with high scores on the ProQoL scale. Unlike the study by Nanjundeswaraswamy et al. [29], which evaluated QoWL at two different time points, before and during the pandemic, the studies by Daşbilek et al. [30] and You [28] did not offer a temporal comparison, preventing a direct assessment of the impact the pandemic may have had on these aspects of professional QoL. However, findings from previous studies [47] emphasize that the situation for nursing professionals was already unfavorable before the pandemic, with high levels of stress and emotional exhaustion, conditions that were exacerbated by the extreme workloads and lack of resources characteristic of the health crisis [48]. This context suggests that the pandemic did not generate an entirely new phenomenon but rather amplified a pre-existing issue that was already affecting nurses in various regions. Consequently, it is essential to consider the implementation of psychological support policies and stress management programs that not only address exceptional situations but are structurally and preventively integrated into daily work practices. This is crucial to improving nurses’ QoWL and ensuring healthy and sustainable work environments in the long term.

### 4.3. Factors Associated with QoL

The third axis addresses the factors associated with QoL. Through the review of factors related to the QoL of nursing professionals, several key determinants impacting their well-being are identified. These factors range from sociodemographic aspects to working and health conditions, with significant variations across studies. The COVID-19 pandemic has intensified many of these issues, highlighting the urgent need to review and reform institutional policies to improve the working conditions and health of nursing professionals.

In this regard, sociodemographic factors play an essential role in the QoL of nursing professionals [49]. Concerning gender, the studies reviewed consistently show that women experience higher levels of burnout and STS compared to men, as observed by You [28] and Daşbilek et al. [30]. This gender difference may reflect the additional burdens women face in both their personal and professional lives, leading to greater levels of emotional exhaustion. This finding highlights the need for institutional policies that address gender inequalities in the workplace, providing adequate support for nurses and improving their working conditions to reduce stress and emotional fatigue.

Educational level is also associated with the QoL of nursing professionals. The studies by You [28] and Daşbilek et al. [30] indicate that nurses with a postgraduate education report higher levels of burnout and STS compared to those with undergraduate or associate degrees. This finding suggests that while professionals with more education may have more responsibilities and demanding work, they may also experience higher job-related stress due to the higher expectations associated with their educational level. This phenomenon underscores the importance of establishing emotional and psychological support mechanisms for these professionals, ensuring that their competencies are adequately recognized without overloading them with expectations.

Regarding family, living with a partner and having support in childcare are positively associated with a better QoL, as observed in the studies by Antolí-Jover et al. [26]. These results suggest that professionals who have a family support network are better equipped to manage the emotional and physical demands of the profession. On the other hand, having children, although associated with greater compassion satisfaction, is also linked to higher levels of burnout, which may reflect the tension between work demands and family responsibilities. Policies promoting WLB, such as flexible schedules or access to childcare and support services, could be key to improving the QoL of nursing professionals [44].

Occupational factors equally determine the QoL of nursing professionals. The reviewed research indicates that working conditions, shift type, and work environment significantly influence nurses’ well-being. Fixed shifts are associated with lower levels of burnout and STS compared to rotating shifts, as noted by You [28] and Al-Hammouri et al. [27]. In contrast, Antolí et al. [26] suggest that shifts may be associated with a better perception of HRQoL, as they allow for a greater degree of flexibility for some professionals to balance their work and personal responsibilities. Therefore, an effective organizational policy should be inclusive and adaptable, allowing all nursing professionals the opportunity to balance their work and personal responsibilities in a healthy way, improving their QoL, and reducing the negative effects of stress and burnout on their job performance and overall well-being.

Nursing professionals’ work environment, relationships with their colleagues, and organizational culture are also key factors that impact their QoL. The studies by Nanjundeswaraswamy et al. [29] indicate that a positive work environment and a supportive organizational culture are associated with a better QoL. These findings underscore the need for healthcare institutions to foster healthy work environments, promoting collaboration among colleagues and mutual support. Institutional policies should focus on improving communication, cooperation, and the overall well-being of staff to mitigate the negative effects of job stress.

Moreover, an adequate WLB has been identified as one of the most influential factors on nurses’ QoL. The studies reviewed suggest that a positive WLB has favorable effects on QoL, whereas conflict between both domains significantly reduces professional well-being. Specifically, the study by You [28] highlighted that a good WLB not only reduced burnout but also increased compassion satisfaction and decreased STS, emphasizing the importance of this balance for the mental and emotional health of nurses. In this regard, it has been observed that work–family conflict, particularly when work demands interfere with family responsibilities, leads to increased levels of stress and emotional exhaustion, as reflected in research such as that of Al-Hammouri et al. [27] and Daşbilek et al. [30]. These conflicts, both from work to family and from family to work, are associated with greater pressure on professionals, negatively affecting their QoL.

However, it is also important to highlight that not all interactions between work and family are negative. This is reflected in the study conducted by Antolí et al. [26], who suggest that in some cases, there may be a positive synergy in which the relationship between work and personal life contributes to greater satisfaction and overall well-being. This finding suggests that a comprehensive approach to WLB should consider both the negative and positive aspects of this interaction in order to provide a more complete view of the factors that affect QoWL.

Finally, health status has a direct impact on the QoL of nursing professionals. You [28] found that those with poorer physical and mental health reported higher levels of burnout, lower levels of compassion satisfaction, and higher levels of STS. This relationship between health and QoL underscores the importance of healthcare institutions implementing strategies to improve their employees’ physical and mental health, such as wellness programs, psychological counseling, and physical health support.

The findings of this review suggest that healthcare institutions should consider a comprehensive reform of labor policies that address not only the working environment conditions but also the balance between work and personal life.

### 4.4. Limitations

The study has several limitations. First, the lack of geographical diversity: of the five studies selected, only one was conducted in Europe, while the other four were conducted in Asia. However, although most of the studies come from the Asian continent, there are important cultural differences between them. Despite these variations, the results obtained seem to be consistent, suggesting that the conclusions could be valid at a global level.

No studies from other regions of the world were identified, which limits the generalizability of the results and reflects an important gap in the current literature on WLB in the nursing field during the pandemic. This suggests that the experiences and challenges in other regions of the world, such as Latin America, Africa, and North America, have not been sufficiently explored. In addition, the review included a total of only five studies, which may limit the robustness of the conclusions obtained. Such a small number of investigations makes it difficult to extract clear patterns and to carry out more detailed comparative analyses between different regions, work contexts, and health systems.

The variability in the measurement of HRQoL and QoWL represents another limitation, since the studies reviewed used different instruments to measure HRQoL and QoWL. This disparity in methodological approaches complicates the direct comparison of results and creates the need to develop or implement unified tools that allow for a comprehensive and more accurate assessment of these aspects that have been identified thanks to this study.

Another limitation that was found is the focus on negative aspects. Although some studies mention positive influences of work on family life, most studies focus on conflicts and tensions between these two areas. This can lead to a biased view and does not fully reflect the possible beneficial interactions between both contexts, limiting a more balanced understanding of WLB. These limitations highlight the need for future broader, more diversified and comparative research that addresses nurses’ WLB in different contexts.

## 5. Conclusions

This study has synthesized the available evidence on the WLB and QoL of nursing professionals during the COVID-19 pandemic, highlighting the significance of these factors in the health and well-being of healthcare workers. The results indicate that the pandemic has exacerbated pre-existing challenges related to WLB, particularly among professionals with rigid and extended working hours, which increases their levels of stress and emotional burden, negatively affecting their QoL. In this context, labor policies and institutional support must evolve to adapt to new realities, promoting a healthier balance between work and personal life.

The findings also emphasize that, although the global health crisis led to a widespread decrease in nurses’ QoL, flexibility in work schedules and adequate support for work–family reconciliation can mitigate adverse effects. The presence of social and family support networks is positively associated with a better QoL, underscoring the importance of institutional policies that promote gender equity and WLB. In general, the pandemic not only revealed pre-existing shortcomings in working conditions but also highlighted the urgent need for an integrated approach that addresses both the emotional and physical aspects of nursing professionals’ well-being.

Finally, the results suggest that implementing flexible labor policies, psychological support programs, and a collaborative work environment are essential for improving nurses’ QoL, reducing stress, and preventing burnout. These measures are crucial for ensuring the health and well-being of nursing professionals and, therefore, maintaining the quality of care they provide to patients.

## Figures and Tables

**Table 1:** MMAT methodological quality for descriptive quantitative studies.

Studies	S1	S2	4.1	4.2	4.3	4.4	4.5	%
Antolí et al. (2024) [26]	S	S	S	S	S	S	S	100%
Nanjundeswaraswamy et al. (2023) [29]	S	S	S	S	S	NS	S	80%
Lorber et al. (2022) [31]	S	S	S	NS	NO	NS	S	40%
Al-Hammouri et al. (2023) [27]	S	S	S	S	S	S	S	100%
You, (2021) [28]	S	S	S	S	S	S	S	100%
Daşbilek et al. (2022) [30]	S	S	S	S	S	NS	S	80%

S (Yes); N (No); NS (I don’t know); S1 (Are there clear research questions?); S2 (Do the data collected allow the research questions to be addressed?); 4.1 (Is the sampling strategy relevant to address the research question?); 4.2. (Is the sample representative of the target population?); 4.3. (Are the measures appropriate?); 4.4. (Is the risk of non-response bias low?); 4.5. (Is the statistical analysis appropriate to answer the research question?). Source: Own elaboration.

**Table 2:** Description of the selected articles.

Author, Country, and Year	Design	Sample	QoL and WLB Results
Antolí et al., Spain, 2024 [26]	Cross-sectional descriptive study	N: 305	Instrument EQ-5D [32] (M ± S.D): EQ-5D Index: 0.820 ± 0.154 EQ-VAS: 74.56 ± 15.735 Instrument SWING [33] (M ± S.D): Negative Work–Family: 1.24 ± 0.519 Negative Family–Work: 0.46 ± 0.443 Positive Work–Family: 1.44 ± 0.634 Positive Family–Work: 1.95 ± 0.693
Nanjundeswaraswamy et al., India, 2023 [29]	Cross-sectional descriptive study pre- and post-pandemic	N: 405 (pre: n = 209) (post: n = 196)	Custom instrument (QoWL, regression equation) [29]: Pre-pandemic:- QoWL 4.36 COVID-QoWL: 3.35; *p* = 0.027 Custom instrument WLB [29] (M ± S.D): Pre-pandemic WLB: 4.18 ± 0.616 COVID-WLB: 3.40 ± 0.482
Al-Hammouri et al., Jordan, 2023 [27]	Cross-sectional descriptive study	N: 216	Instrument WRQoL [34] (M ± S.D) 3.03 ± 0.75 Instrument WAFCS [34] (M ± S.D): Work–Family Conflict: 16.31 ± 6.17 Family–Work Conflict: 23.6 ± 6.91
You, South Korea, 2021 [28]	Cross-sectional descriptive study	N: 208	Instrument ProQoL [35] (M ± S.D): BO: 2.96 ± 0.49 CS: 2.89 ± 0.64 STS: 2.58 ± 0.59 Instrument WLBS [36] (M ± S.D): WLB: 2.43 ± 1.02
Daşbilek et al., Turkey, 2022 [30]	Cross-sectional descriptive study	N: 507	Instrument ProQoL [35] (M ± S.D): BO: 28.58 ± 5.70 CS: 32,953 ± 6.169 STS: 27.19 ± 6.54 Instrument WLBS [37] (M ± S.D): Work–Family Conflict: 20.282 ± 2.909 Family–Work Conflict: 18.598 ± 4.504

Source: Own elaboration. BO: Burnout; CS: Compassion Satisfaction; STS: Secondary Traumatic Stress.

**Table 3:** Factors associated with quality of life (QoL).

Author, Country and Year	Factors Associated with QoL	Outcomes		
Antolí et al., Spain 2024 [26]		EQ-5D Index:	EQ-5D VAS:	
	Negative work–family	ß = −0.399; *p* < 0.05	ß = −0.337; *p* < 0.05	
	Number of children	ß = −0.146; *p* < 0.05		
	Paid caregiver support	ß = 0.149; *p* < 0.05	ß = 0.18; *p* < 0.05	
	Living with a partner	ß = 0.174; *p* < 0.05	ß = 0.16; *p* < 0.05	
	Rotating shift	ß = 0.158; *p* < 0.05		
	Positive work–family		ß = 0.218; *p* < 0.05	
Nanjundeswaraswamy et al., India, 2023 [29]		Pre-pandemic QoWL	COVID:-QoWL	
	Work condition	ß = 0.209; *p* = 0.004		
	Work environment	ß = 0.160; *p* = 0.025		
	Compensation reward	ß = 0.200; *p* = 0.002		
	Organizational culture	ß = 0.235; *p* = 0.001		
	Work life balance		ß = 0.251; *p* = 0.001	
	Relationship among co-workers		ß = 0.171; *p* = 0.011	
	Career development		ß = 0.196; *p*= 0.004	
Al-Hammouri et al., Jordan, 2022 [27]		QoWL		
	Family–work conflict	r = −0.20; *p* = 0.01		
	Work–family conflict	r = −0.49; *p* = 0.01		
	Shifts	Changing shifts 2.83 Fixed shift 3.23 (*p* = 0.004)		
You, South Korea, 2021 [28]		BO	CS	STS
	Work–life balance	ß = −0.68; *p* < 0.001	ß = 0.42; *p* < 0.001)	ß = −0.56; *p* < 0.001
	Work shift	Shift 3.00 ± 0.50 Day fixed 2.81 ± 0.42; (*p* = 0.017)	Shift 2.83 ± 0.67; Day fixed 3.06 ± 0.52; (*p* = 0.027)	
	Health status	Good 2.57 ± 0.40; Fair 3.05 ± 0.426; Bad 3.30 ± 0.57 *p ≤* 0.001	Good 3.21 ± 0.63; Fair 2.81 ± 0.56; Bad 2.55 ± 0.87 *p ≤* 0.001	Good 2.22 ± 0.59; Fair 2.65 ± 0.50; Bad 3.04 ± 0.58 *p ≤* 0.001
	Gender	Women 2.97 ± 0.48; Men 2.59 ± 0.61; *p* = 0.029		Women 2.60 ± 0.57; Men 2.06 ± 0.79; *p* = 0.011
	Educational level			College 2.69 ± 0.61 University 2.53 ± 0.58 ≥ Graduate school 3.07 ± 0.24 *p* = 0.011
	Job role			Staff nurse 2.58 ± 0.60; Charge nurse 2.58 ± 0.53; Head nurse 2.55 ± 0.53; *p* = 0.02
Daşbilek et al., Turkey, 2022 [30]		BO	CS	STS
	Gender	Women 28.945 ± 5.686; Men 27.781 ± 5.664; *p* = 0.032	Women 33.677 ± 6.046; Men 31.381 ± 6.161; *p ≤* 0.001	Women 27.640 ± 6.543; Men 26.213 ± 6.456; *p ≤* 0.022
	Educational level	(1) College 26.500 ± 7.911; (2) University 28.098 ± 4.760; (3) ≥Graduate school 28.960 ± 5.484; *p* = 0.011 (3 > 1 (*p* < 0.05))		(1) College 24.300 ± 8.835; (2) University 27.390 ± 6.085; (3) ≥ Graduate school 27.531 ± 6.199; *p* = 0.004 (2 > 1, 3 > 1 (*p* < 0.01))
	Professional seniority	(1) <1 year 29.989 ± 6.367; (2) 1–4 years 28.698 ± 5.344; (3) 5–9 years 26.781 ± 5.823; (4) ≥10 years 30.178 ± 4.477; *p* < 0.001 (4 > 2, 1 > 3, 2 > 3, 4 > 3 (*p* < 0.001)	(1) <1 year 33.602 ± 5.941; (2) 1–4 years 32.007 ± 6.020; (3) 5–9 years 32.041 ± 5.876; (4) ≥10 years 35.307 ± 6.445; *p* < 0.001 (1 > 2, 4 > 2, 1 > 3, 4 > 3 (*p* < 0.05)	(1) <1 year 29.148 ± 6.793; (2) 1–4 years 26.758 ± 5.911; (3) 5–9 years 25.402 ± 6.736; (4) ≥10 years 29.109 ± 5.980; *p* < 0.001 (1 > 2, 4 > 2, 1 > 3, 4 > 3 (*p* < 0.05))
	Job tenure in the current position	(1) <1 year 28.449 ± 6.267; (2) 1–4 years 28.674 ± 5.591; (3) 5–9 years 27.858 ± 5.222; (4) ≥10 years 30.949 ± 4.255; *p* = 0.032 (4 > 1, 4 > 2, 4 > 3 (*p* < 0.05))	(1) <1 year 32.324 ± 6.017; (2) 1–4 years 32.785 ± 6.059; (3) 5–9 years 33.358 ± 6.163; (4) ≥10 years 35.282 ± 6.920; *p* = 0.045 (4 > 1, 4 > 2 (*p* < 0.05))	(1) <1 year 27.119 ± 6.941; (2) 1–4 years 26.913 ± 6.017; (3) 5–9 years 26.542 ± 6.225; (4) ≥10 years 30.718 ± 7.030; *p* = 0.005 (4 > 1, 4 > 2, 4 > 3 (*p* < 0.01))
	Having children	Yes 29.489 ± 5.359; No 28.050 ± 5.831; *p* = 0.006	Yes 33.844 ± 6.627; No 32.436 ± 5.837; *p* = 0.013	

## Data Availability

The original contributions presented in the study are included in the article, further inquiries can be directed to the corresponding authors.

## Public Involvement Statement

There was no public involvement in any aspect of this research.

## Guidelines and Standards Statement

This manuscript was drafted using PRISMA (Preferred Reporting Items for Systematic Reviews and Meta-Analyses for Protocols) guidelines.
